# Leveraging Resources to Remove a Taser Barb Embedded in Bone: Case Report

**DOI:** 10.5811/cpcem.2021.10.54196

**Published:** 2022-01-28

**Authors:** Lauren Willoughby, Kelee Peyton, Diane Gorgas, Simiao Li-Sauerwine

**Affiliations:** The Ohio State University, Department of Emergency Medicine, Columbus, Ohio

**Keywords:** conducted electrical weapon, taser, foreign body removal, fulcrum technique, case report

## Abstract

**Introduction:**

Conducted electrical weapons, commonly known by their proprietary eponym, TASER, are frequently used by law enforcement. A review of the literature yielded descriptions of taser barb removal from soft tissue and surgical intervention for barbs lodged in sensitive areas such as the eye and head, but not from other osseous sites.

**Case Report:**

We report the case of a 30-year-old male transferred from another hospital with a taser dart embedded in his clavicle. Prior attempts at bedside removal had been unsuccessful. We describe bedside removal of the taser barb from bone using local anesthesia and simple fulcrum technique.

**Conclusion:**

We describe a novel fulcrum technique for removal of a taser dart embedded in bone. This is a reasonable technique to attempt in patients with involvement of superficial osseous structures to avoid operative intervention.

## INTRODUCTION

The conducted electrical weapon was invented in 1974 by Jack Cover, a former National Aeronautics and Space Administration researcher. He named the device TASER (Axon Enterprise, Inc., Scottsdale, AZ) after the 1911 children’s science fiction book *Tom Swift and His Electric Rifle*. These devices have remained popular as a less-lethal tool in law enforcement and self-defense when compared to traditional firearms. When activated, a taser fires two darts from a cartridge via compressed nitrogen canister at 55 meters per second and has a range of 15–35 feet. Each dart is made of a fish-hook barb on a metal shaft attached to a metal and plastic cylinder that is then connected to the taser by thin copper wires. An electrical pulse can then be delivered between the darts, causing contraction of skeletal muscle and incapacitating the target.^1^

Although the size of the dart (9.5 millimeters [mm] long, 0.8 mm diameter) often prevents clinically significant depth of penetration, the fish-hook barb is designed so that darts will lodge in place upon impact. Three methods for removal were described previously by Koscove in 1985: 1) grasp the wire or dart firmly and pull it out with in-line traction; 2) cover the barb with a 16 G needle and then withdraw the dart in a method similar to fish-hook removal; and 3) prep the skin and administer local anesthetic prior to cutting down to the barb and removing it through the incision.^2^

We were unable to find any current literature comparing or documenting the efficacy of these strategies, although one article did advise in-line traction as the first step.^3^ However, the methods described by Koscove are intended specifically for the removal of darts embedded in skin or soft tissue. For darts embedded in other sensitive regions (most often defined as the face, groin, breast, eye, or head), operative or specialist intervention is often recommended.^4^ To our knowledge, no bedside strategy for the removal of taser darts embedded in bone has been described.

## CASE REPORT

A 30-year-old male presented with a taser dart embedded in his left clavicle. He was initially seen at another hospital and multiple attempts were made to remove the dart after administration of local anesthetic (1% lidocaine without epinephrine) and manual in-line traction along the axis of the dart, perpendicular to the clavicle. These attempts had been unsuccessful despite the patient tolerating the procedure well and reporting minimal discomfort. He was ultimately transferred to our institution for further management and possible surgical evaluation.

**Image 1:**
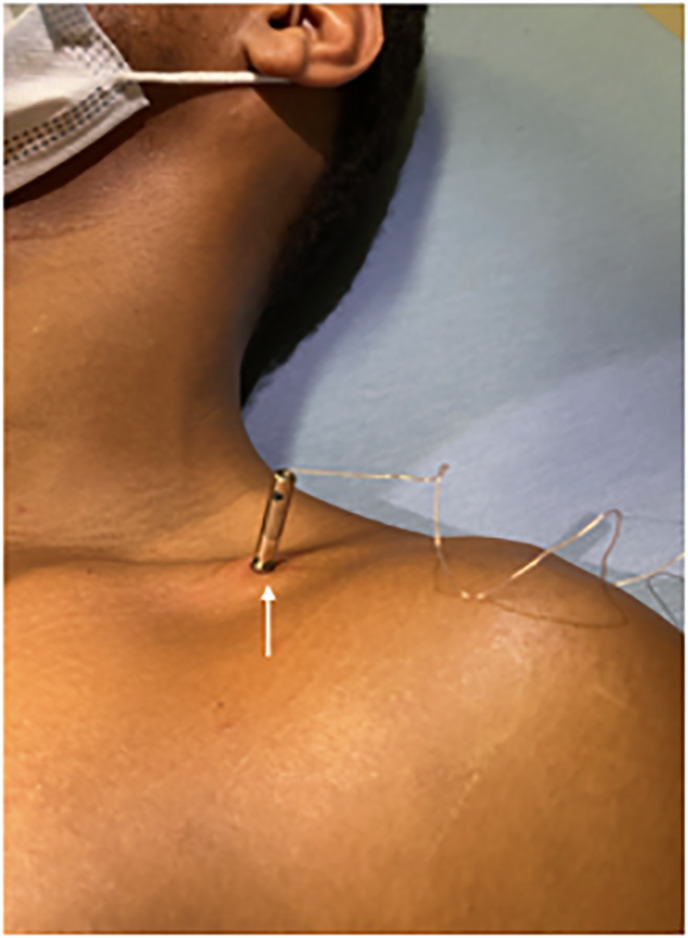
Taser barb embedded in the left, anterior, mid clavicle (white arrow).

On arrival to the emergency department, the patient’s physical exam showed the taser barb still lodged in his clavicle but with no active bleeding or other soft tissue injury (Image 1). Additionally, no neurovascular deficits were identified. A repeat radiograph of the clavicle showed the barb embedded approximately 4 mm into the clavicle with a small hook deformity of the barb tip, but no associated fractures. To avoid surgery and with consent of the patient, bedside removal was again attempted.

**Image 2:**
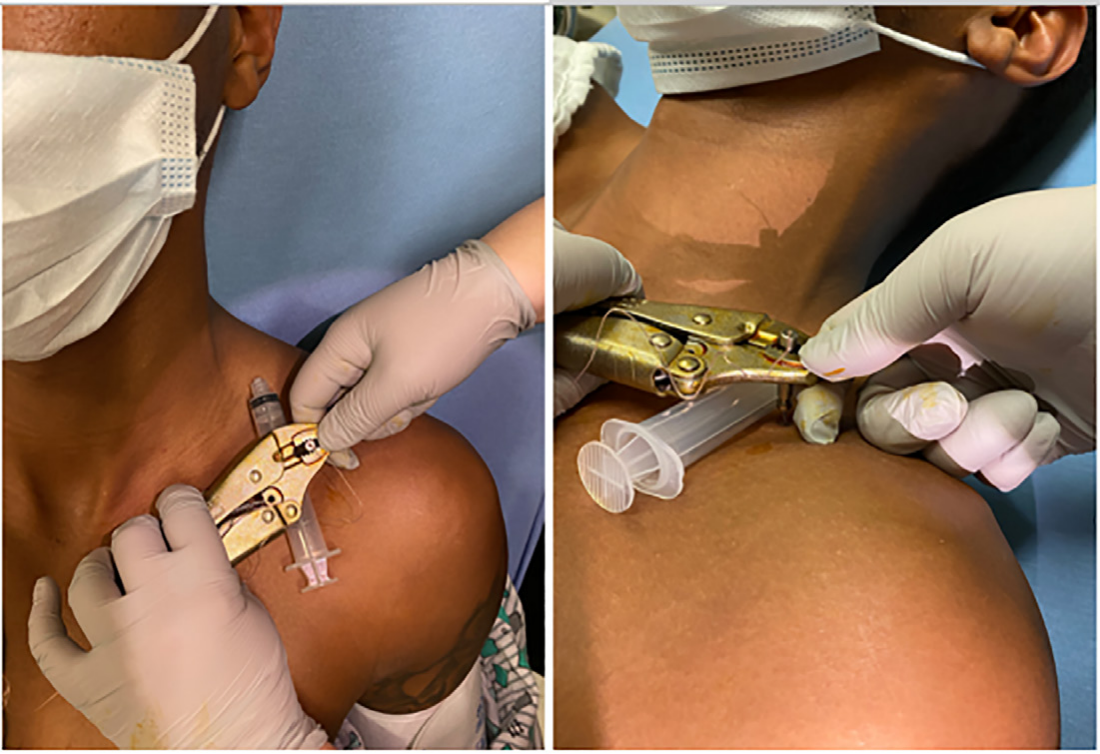
Vise-grip locking pliers were used to grasp the metal cylinder, and a 10-cc* syringe was placed below the pliers to act as a fulcrum when downward pressure was applied to the plier handles. *cc, cubic centimeter.

Prior to the procedure, ketorolac 15 milligrams was administered intravenously. The surrounding skin was prepped with a betadine solution. Anesthesia was achieved via local infiltration of a 50/50 mixture of 1% lidocaine and 0.5% bupivacaine without epinephrine at the periosteum and along the track of the barb. The patient reported excellent pain relief and complete anesthesia at the site of the clavicular foreign body. A pair of vise-grip locking pliers was then used to grasp the metal cylinder, and a 10-cubic centimeter (cc) syringe was placed under the pliers, adjacent to the cylinder. The syringe was used as a fulcrum to lever the barb out of the bone (Image 2). The taser barb was easily removed and the patient tolerated the procedure with no bleeding or additional trauma noted to the surrounding soft tissues. A post-procedure radiograph showed no fracture but did demonstrate a small retained foreign body of the taser-barb tip in the clavicle.

**CPC-EM Capsule**

What do we already know about this clinical entity?

*Bedside removal of taser barbs from soft tissue sites and surgical intervention for barbs lodged in sensitive locations have been previously described*.

What makes this presentation of disease reportable?

*We describe bedside removal of a taser barb percutaneously embedded in an osseous site*.

What is the major learning point?

*An alternative to in-line traction is the use of a syringe as a fulcrum, which leverages clinician effort when removing a taser dart from an osseous site*.

How might this improve emergency medicine practice?

*Using the fulcrum method allows for greater likelihood of success for bedside removal of a taser barb and may eliminate the need for operative management*.

Local wound care was administered, and a seven-day course of cephalexin was prescribed for prophylaxis in the setting of penetrating trauma involving the bone. The patient’s tetanus status was confirmed to be up to date. He was then discharged in stable condition.

## DISCUSSION

Currently there is a paucity of literature addressing strategies for removal of taser barbs. Most reports focus on cases of ocular and cranial penetration, which are relatively rare and almost always require immediate specialist intervention.^3^ Due to lack of available data, it is not clear how often emergency physicians remove these barbs from patients. One study in Salt Lake City, Utah, identified 648 emergency medical service (EMS) activations over five years for the indication of taser barb removal, indicating that this is a relatively rare procedure with a prevalence of 4.55 per 1,000,000 EMS activations.^5^ However, it should also be noted that there is significant regional variance in removal policies. While some EMS agencies have protocols for dart removal, others prohibit emergency medical technicians from doing so in the field. As the use of tasers has become more widespread over the years, there is an ongoing need for emergency physicians to be trained in taser-related injuries.

In our case, the traditional in-line traction method had been attempted previously and was unsuccessful, likely due to depth of osseous penetration. For this presentation, it was not practical to use the removal methods described by Koscove. Ultimately, our use of the 10-cc syringe as a fulcrum allowed us to gain sufficient leverage on the dart for removal. This had the benefit of averting the need for operative intervention and potential associated risks of surgery. Additionally, the administration of local and periosteal anesthetic in conjunction with intravenous analgesics was found to be sufficient to achieve pain control and eliminated the need for conscious sedation.

While there were no immediate complications from our procedure, we identified several potential considerations in choosing this method. We recommend assessing the appropriateness of the location over which the syringe will be placed, to decrease the likelihood of injury to underlying structures or exacerbating previously existing injuries such as fractures. Additionally, care must be taken to protect the free hand stabilizing the dart to prevent the clinician from being injured by the barb as it is pulled free of the patient. Our patient’s tetanus status was up to date, but out of an abundance of caution we decided to administer prophylactic antibiotics given the presence of a retained foreign body and penetrating injury to the bone. Unfortunately, our patient was lost to follow-up; so it is unclear what his ultimate outcome was and whether secondary infection occurred.

## CONCLUSION

We describe an alternative method for the removal of taser darts embedded in bone. This strategy was ultimately effective after the traditional methods of removal by in-line traction proved to be insufficient. While it is no substitute for expert consultation in circumstances where the dart has become embedded in sensitive areas, we feel it is reasonable to attempt in patients with osseous involvement as it may help avoid the risks of operative intervention.
